# Physicians’ perspectives on clinical indicators: systematic review and thematic synthesis

**DOI:** 10.1093/intqhc/mzae082

**Published:** 2024-08-10

**Authors:** Ana Renker-Darby, Shanthi Ameratunga, Peter Jones, Corina Grey, Matire Harwood, Roshini Peiris-John, Timothy Tenbensel, Sue Wells, Vanessa Selak

**Affiliations:** Epidemiology & Biostatistics, University of Auckland, Private Bag 92019, Auckland 1142, New Zealand; Epidemiology & Biostatistics, University of Auckland, Private Bag 92019, Auckland 1142, New Zealand; Service Improvement and Innovation Directorate, Te Whatu Ora—Health New Zealand, Private Bag 93311, Auckland 1640, New Zealand; Adult Emergency Department, Auckland City Hospital, Private Bag 92024, Auckland 1142, New Zealand; Department of Surgery (Emergency Medicine), University of Auckland, Private Bag 92019, Auckland 1142, New Zealand; Epidemiology & Biostatistics, University of Auckland, Private Bag 92019, Auckland 1142, New Zealand; General Practice and Primary Health Care, University of Auckland, Private Bag 92019, Auckland 1142, New Zealand; Epidemiology & Biostatistics, University of Auckland, Private Bag 92019, Auckland 1142, New Zealand; Health Systems, University of Auckland, Private Bag 92019, Auckland 1142, New Zealand; General Practice and Primary Health Care, University of Auckland, Private Bag 92019, Auckland 1142, New Zealand; Epidemiology & Biostatistics, University of Auckland, Private Bag 92019, Auckland 1142, New Zealand

**Keywords:** clinical indicators, physician perspectives, systematic review

## Abstract

Clinical indicators are increasingly used to improve the quality of care, particularly with the emergence of ‘big data’, but physicians’ views regarding their utility in practice is unclear. We reviewed the published literature investigating physicians’ perspectives, focusing on the following objectives in relation to quality improvement: (1) the role of clinical indicators, (2) what is needed to strengthen them, (3) their key attributes, and (4) the best tool(s) for assessing their quality. A systematic literature search (up to November 2022) was carried out using: Medline, EMBASE, Scopus, CINAHL, PsycInfo, and Web of Science. Articles that met all of the following inclusion criteria were included: reported on physicians’ perspectives on clinical indicators and/or tools for assessing the quality of clinical indicators, addressing at least one of the four review objectives; the clinical indicators related to care at least partially delivered by physicians; and published in a peer-reviewed journal. Data extracted from eligible studies were appraised using the Critical Appraisal Skills Programme tool. A thematic synthesis of data was conducted using NVivo software. Descriptive themes were inductively derived from codes, which were grouped into analytical themes answering each objective. A total of 14 studies were included, with 17 analytical themes identified for objectives 1–3 and no data identified for objective 4. Results showed that indicators can play an important motivating role for physicians to improve the quality of care and show where changes need to be made. For indicators to be effective, physicians should be involved in indicator development, recording relevant data should be straightforward, indicator feedback must be meaningful to physicians, and clinical teams need to be adequately resourced to act on findings. Effective indicators need to focus on the most important areas for quality improvement, be consistent with good medical care, and measure aspects of care within the control of physicians. Studies cautioned against using indicators primarily as punitive measures, and there were concerns that an overreliance on indicators can lead to narrowed perspective of quality of care. This review identifies facilitators and barriers to meaningfully engaging physicians in developing and using clinical indicators to improve the quality of healthcare.

## Introduction

Clinical indicators are measures designed to assess and improve the quality of health services. When they seek to support quality improvement efforts by clinicians, it is critical to meaningfully engage with clinicians in the development and monitoring of these indicators [1]. Previous research has found that among clinicians, physicians may be resistant to clinical indicator initiatives, particularly when indicators are designed for the purpose of accountability rather than quality improvement [2]. If physicians are more engaged with indicator development and use, they are more likely to accept and act upon findings from those indicators [1].

Traditionally, there has been a focus on manual audits of patient records as a mechanism for supporting quality improvement. The availability of ‘big data’ (high volumes of diverse electronic data [3]) has the potential to revolutionize clinical engagement with quality improvement activities [4]. Rather than undertaking static, intermittent audits of a subset of patients, big data makes it possible to continuously generate electronic data on clinical indicators to improve performance [5]. Further, techniques such as natural language processing can enhance the clinical relevance of identifiable cohorts by enabling free text as well as structured data to be interrogated systematically [6, 7]. To maximize the extent to which these advances translate into improvements in the quality of care, it is critical that, where indicators are designed to support quality improvement efforts by physicians, meaningful clinical engagement with physicians is obtained in the development and monitoring of clinical indicators.

Previously Jones *et al*. developed a Quality Indicator Clinical Appraisal (QICA) tool to appraise indicators based on key attributes identified through a systematic review and survey of quality of care experts [8]. The QICA tool ‘provides an explicit basis for discussions around indicator selection’ [8]. However, ensuring that physicians will use and act on clinical indicator data also requires consideration of physicians’ perspectives. The objectives of this study were to determine physicians’ perspectives regarding (1) the role indicators play in supporting quality improvement, (2) what is needed to strengthen the ability of indicators to drive improvements in quality, (3) the ‘key’ attributes of an effective indicator, and (4) the best tool(s) for assessing the quality of indicators.

## Methods

The systematic review protocol was registered with the International Prospective Register of Systematic Reviews (PROSPERO CRD42020152496).

### Search strategy

A systematic literature search was carried out using Medline, EMBASE, Scopus, Cochrane, CINAHL, PsycInfo, and Web of Science (searched up to November 2022). The search strategies are provided in Supplementary Appendix S1. One reviewer (A.R.) screened all (deduplicated) titles and abstracts using the inclusion and exclusion criteria. Full texts were then screened by A.R., and included texts were discussed with a co-author (V.S.).

### Inclusion and exclusion criteria

Articles were included if they: reported on data from physicians, either as a group or subgroup; focused on clinical indicators and/or tools for assessing the quality of clinical indicators; reported on physicians’ perspectives on clinical indicators and/or tools for assessing the quality of clinical indicators; related to clinical care at least partially delivered by physicians; were published in a peer-reviewed journal; and addressed at least one of the four objectives from the perspective of physicians.

Articles were excluded if they: reported on data from health professionals, patients and/or family members without any physicians or without separate reporting for physicians; focused on evaluating quality of care, clinical guidelines, models of care or diagnostic criteria; were in a language other than English; were editorial or opinion pieces; or had insufficient data to adequately interpret the results. No time restrictions or methodological restrictions were applied.

### Quality appraisal

As all included citations used qualitative methodologies, we selected the 10-item Critical Appraisal Skills Programme (CASP) qualitative studies tool [9] to assess and enable the quality of each citation to be factored in the review. A.R. reviewed the studies using the CASP checklist and discussed findings with V.S. until consensus was reached.

### Data extraction and thematic synthesis

The method of thematic synthesis was adapted from Thomas and Harden [10] and guided by that detailed by Braun and Clarke [11]. A.R. and V.S. independently screened the results and discussion sections of all included articles to extract data consistent with the inclusion and exclusion criteria for this review (including direct quotes from study participants and author interpretations). Differences between A.R. and V.S. in data inclusion decisions were discussed until consensus was reached. A.R. coded each sentence or phrase with one or more codes using NVivo software [11]. Descriptive themes were inductively derived from codes, which were then grouped into analytical themes answering each of the objectives. Codes were renamed iteratively where relevant or combined if they addressed similar ideas. Themes were discussed between V.S. and A.R. until consensus was reached, and then reviewed and approved by all authors.

### Ethics

Ethics approval was not required as this study is a systematic review of published literature.

## Results

### Study selection

**Figure 1:**
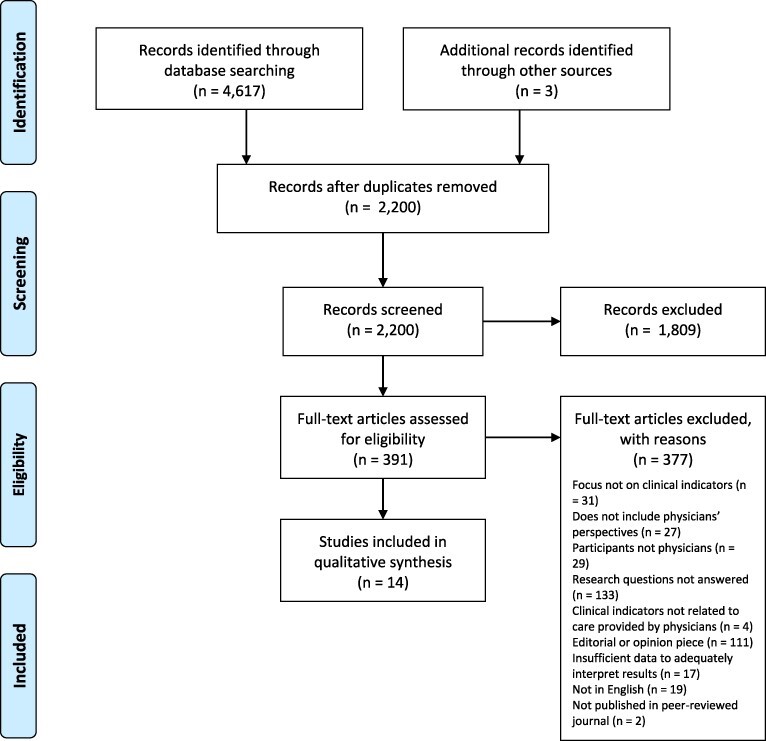
PRISMA flow diagram of study selection.

From 4620 initial citations, a total of 14 studies were included in the study (Fig. 1). These included papers were published between 2000 and 2022 and were based in the United Kingdom, United States, Canada, China and Germany, in teaching hospitals, primary care groups, and an ambulatory care organization (Table 1).

**Table 1.** Summary of included studies.

Study (year)	Aim	Method	Setting	Participants
Ahmed *et al*. (2019)	To explore the views of clinician–scientists and quality improvement experts regarding proposed domains of PCC, and to gain an understanding of current practices and opportunities for measurement of PCC at a healthcare system level.	Semi-structured interviews (*n* = 16)	Canada, USA, UK	Clinician–scientists (*n* = 4), Quality improvement experts (*n* = 12)
Benn *et al*. (2015) Chapter 6: Qualitative Evaluation*	To conduct a quasi-experimental evaluation of the feedback initiative and its effect on quality of anaesthetic care and perioperative efficiency.	Interviews (*n* = 35)	Teaching hospital in London, UK	Consultant anaesthetists (*n* = 24), surgical nursing leads (*n* = 6), perioperative service leads (*n* = 5)
Breidenbach *et al*. (2021)	To identify factors that inhibit of facilitate the usage of PROs for clinical decision-making and monitoring patients in existing structures for oncological care, certified colorectal cancer centres in Germany.	Semi-structured interviews (*n* = 12)	Cancer centres participating in EDIUM study in Germany	Physicians (*n* = 7), psycho-oncologist (*n* = 1), nurses (*n* = 3), physician assistant (*n* = 1)
D’Lima *et al*. (2017)*	To report the experience of anaesthetists participating in a long-term initiative to provide comprehensive personalized feedback to consultants on patient-reported quality of recovery indicators in a large London teaching hospital.	Semi-structured interviews (*n* = 21)	Teaching hospital in London, UK	Consultant anaesthetists (*n* = 13), surgical nursing leads (*n* = 6), theatre manager (*n* = 1), clinical coordinator for recovery (*n* = 1)
Exworthy *et al*. (2003)^a^	To review qualitative findings from an empirical study within one English primary care group on the response to a set of clinical performance indicators relating to general practitioners in terms of the effect upon their clinical autonomy.	Semi-structured interviews (*n* = 52)	Primary care group in southern England, UK	GPs (*n* = 29), practice nurses (*n* = 12), practice managers (*n* = 11)
Gagliardi *et al*. (2008)	To explore patient, nurse, physician, and manager preferences for cancer care quality indicators.	Interviews (*n* = 30)	Two teaching hospitals, Canada	Surgeons (*n* = 2), radiation oncologists (*n* = 2), medical oncologist (*n* = 1), nurses (*n* = 5), managers (*n* = 5), patients (*n* = 15)
Gill *et al*. (2012)	To explore the perspectives of general practitioners on the introduction of child-specific quality markers to the UK’s Quality Outcomes Framework.	Semi-structured interviews (*n* = 20)	Five Primary Care Trusts, England	GPs (*n* = 20)
Gray *et al*. (2018)	To explore the role that metrics and measurement play in a wide-reaching ‘Lean’-based continuous quality improvement effort carried out in the primary care departments of a large, ambulatory care healthcare organization.	Semi-structured interviews (*n* = 130)	Large, multispecialty, ambulatory care organization, USA	Primary care physicians (# of participants not disclosed)
Hicks *et al*. (2021)	To identify all available patient-reported outcome measures relevant to diseases treated by vascular surgeons and to evaluate vascular surgeon perceptions, barriers to widespread implementation, and concerns regarding PROs.	Focus groups (# of focus groups not disclosed)	Society for Vascular Surgery, USA	Society for Vascular Surgery members (# of participants not disclosed)
Litvin *et al*. (2015)	To systematically solicit recommendations from Meaningful Use exemplars to inform Stage 3 Meaningful Use clinical quality measure requirements.	Focus groups (*n* = 3)	A national Electronic Health Record-based primary care practice-based research network, USA	General internists (*n* = 5) internal medicine/paediatric physicians (*n* = 2), family medicine physicians (*n* = 16)
Maxwell *et al*. (2002)	To investigate the acceptability among general practitioners of a patient-completed post-consultation measure of outcome and its use in conjunction with two further quality indicators: time spent in consultation and patients reporting knowing the doctor well.	Focus groups (*n* = 7)	Oxford, Coventry, London, and Edinburgh, UK	GPs (*n* = 46)
Rasooly *et al*. (2022)	To understand the current state of quality and performance measurement in primary diabetes care, and the facilitators and barriers to their implementation.	Interviews (*n* = 26)	Tertiary hospitals CHCs in Shanghai, China	Patients (*n* = 12), family doctors (*n* = 3), endocrinologists (*n* = 2), CHC managers (*n* = 4), policymakers (*n* = 5)
Van den Heuvel *et al*. (2010)	To describe and explore the views of German general practitioners on the clinical indicators of the Quality and Outcomes Framework	Focus groups (*n* = 7)	North-western part of Germany	GPs (*n* = 54)
Wilkinson *et al*. (2000)^a^	To investigate reactions to the use of evidence-based cardiovascular and stroke performance indicators within one primary care group.	Semi-structured interviews (*n* = 29)	Fifteen practices from a primary care group in southern England	GPs (*n* = 29)

CHC,  community healthcare centre, GP, General Practitioners, PCC, patient-centred care, PRO, patient-reported outcome, *Articles report on the same study.

a Articles report on the same study but retained to incorporate potential differing interpretations of the data.

### Quality assessment

The articles used qualitative methodology (either interviews or focus groups). The CASP quality assessment revealed that most included studies met most quality criteria (Supplementary Appendix S2). All studies provided clear description of findings. However, there were some methodological limitations with several studies. Nine of 14 articles did not discuss ethical issues with many failing to report ethics approval of the data collection. The relationship between researchers and participants was not adequately discussed in 11 studies, 2 articles did not specify the research aim, and several others failed to report the participant recruitment strategy and only included a limited discussion of how data were analysed.

### Objectives and themes

Data from included studies addressed the first three objectives but no articles addressed the fourth objective.The themes for each objective are described below, and summarized in Table 2.

**Table 2.** Objectives and themes.

Objective	Themes
What is the role of clinical indicators in supporting quality improvement?	Show where changes need to be made
	Motivate physicians to improve quality of care
	Increase physicians’ accountability
	Can encourage myopic quality improvement
	Should be used by physicians, not government or the public
	Should not be used punitively
What is needed to strengthen the ability of indicators to drive improvements in quality?	Support and participation of physicians in their development
	Recording data should be straightforward
	Feedback delivered in a way that is helpful for physicians
	Availability of sufficient resource for quality improvement
	Quality improvement requires working together
	Incentives have advantages and disadvantages
Key attributes of effective indicators	Target the most important areas for quality improvement
	Consistent with good medical care
	Within physicians’ control
	Reliable
	Consider patient-reported measures alongside

### What is the role of clinical indicators in supporting quality improvement?

#### Shows where changes need to be made

Physicians noted that a key role of clinical indicators was their ability to illuminate specific areas of care requiring change. In many cases, physicians stated that it was only through clinical indicators that they received regular feedback on the quality of their care. Physicians appreciated the objective assessment of quality that clinical indicators provided, as opposed to intuiting where care may require improvement. Physicians also thought that clinical indicators could facilitate up-to-date, evidence-based care, provided that the indicators were based on best practice.

#### Motivate physicians to improve quality of care

Physicians commented on two ways in which clinical indicators motivated efforts to improve quality of care: first, seeing the clinical indicator feedback was often a prompt for physicians to take action on quality improvement. Physicians expressed that it was difficult to ignore this type of objective feedback. Second, clinical indicator feedback showing improvements in care motivated physicians, as it demonstrated tangible evidence of how quality improvement could translate into improved outcomes. Many physicians also thought that engaging in quality improvement was part of being a ‘good’ physician.

#### Increase physicians’ accountability

Physicians thought that measuring quality using clinical indicators would make them more accountable for the quality of their care. Some were concerned however that clinical indicators could be used by their organization for performance management, and they feared a loss of autonomy in their practice.

#### Can encourage myopic quality improvement

Physicians were concerned that clinical indicators could lead to a myopic view and produce unintended consequences. They commented that many of the ‘softer’ aspects of quality were difficult to quantify using indicators and risked being side-lined in favour of areas of care more easily quantified. Physicians were concerned that using clinical indicators may distract them from providing more holistic, patient-centred care. Overall, physicians stressed that clinical indicators should be a means to good care, not an end in themselves.

#### Should be used by physicians, not government or the public

Physicians stressed that clinical indicators should be used by physicians for the purpose of quality improvement, not by government or the public. They emphasized the potential for indicators to be misinterpreted by those outside the profession and were worried about being held accountable for measures they could not influence. Physicians also highlighted the tensions between their own priorities for quality improvement and the priorities of government or their organization. They thought that government or organization management were more likely to prioritize productivity and efficiency over the quality of patient care, and were worried that clinical indicators could entrench these priorities.

#### Should not be used punitively

Physicians thought that clinical indicators could either be employed in a ‘soft’ manner to encourage quality improvement or a ‘hard’ manner where poor performance would be criticized or punished. They stressed that this punitive approach would only isolate physicians and was unlikely to improve the quality of care.

### What is needed to strengthen the ability of clinical indicators to drive improvements in quality?

#### Support and participation of physicians in their development

Physicians thought that clinical indicators were more likely to drive improvements in quality if they had the support of clinicians. Physicians were more inclined to use the indicators to make changes to their practice if they understood their purpose and agreed with the measures. They suggested that one way of ensuring their buy-in was to involve them in the development of clinical indicators.

#### Recording data should be straightforward

Physicians thought that recording data for clinical indicators could lead to an unmanageable increase in their workload and may require additional support staff. They suggested that recording indicator data should be integrated into their workflow and automated where possible.

#### Feedback delivered in a way that is helpful for physicians

Physicians had several suggestions for useful ways to deliver clinical indicator feedback. They wanted indicator feedback delivered in a manner that was visually appealing and easy to interpret—most suggested the use of charts rather than tables. Comparison feedback between departments, practices, or individual physicians was also considered useful. Physicians found it helpful to see patterns over time in their feedback. They also highlighted that the timing of feedback was important and should be aligned with appropriate interventions to improve quality.

#### Availability of sufficient resource for quality improvement

Physicians stated that sufficient resources were required for both the use of clinical indicators and subsequent improvements in quality. Physicians also emphasized that they needed sufficient time and resources to reflect on their practice and makes any changes to respond to indicator feedback and improve quality.

#### Quality improvement requires working together

Physicians emphasized that measuring quality of care was not enough to improve quality—it was also crucial that they had support to translate feedback into quality improvement. Most importantly, physicians wanted clinical indicator feedback to be linked to a clear action for improvement. They also suggested that quality improvement needed to happen as a team.

#### Using incentives has advantages and disadvantages

Physicians thought that while tying incentives to clinical indicators could accelerate quality improvement, there was also the potential for unintended consequences and ‘gaming’ the system.

### What are the key attributes of effective indicators?

#### Target the most important areas of care for quality improvement

Physicians thought that the number of clinical indicators should be limited and only cover the most important areas of care. In particular, physicians suggested a focus on diseases where improved care can have a substantial impact, or a focus on especially high-risk patients. Technical process indicators were also suggested as an important aspect of care to measure. Physicians were generally resistant to productivity-oriented indicators.

#### Consistent with good medical care

Physicians thought it was important for clinical indicators to be evidence-based and to reflect best practice. They felt that indicators must be consistent with other policies and guidelines, and indicators should not contradict each other.

#### Within physicians’ control

Physicians thought it was important that clinical indicators measured aspects of care that were within their control. This was particularly important if indicators were tied to incentives or used punitively. Despite many physicians agreeing that outcome indicators measured what was ultimately important, they also expressed concern that outcomes were often affected by factors outside of physicians’ control.

#### Reliable

Physicians commented on several important attributes that would increase their trust in clinical indicators being able to drive improvements in quality. Physicians thought there were several important attributes that made a clinical indicator reliable and hence trustworthy. They stated that clinical indicators should be of high quality, valid, precise, technically specific, clearly defined, and only require information that could be measured accurately.

#### Consider patient-reported measures alongside

Physicians agreed that there was a role for patient-reported outcome measures in driving quality improvement. Patient-reported outcome measures and patient experience indicators were seen as representing one aspect of quality that was important to consider. However, physicians also recommended that such measures should be considered alongside other clinical indicators. They also thought that some aspects of patient experience are subjective and therefore less helpful for quality improvement.

## Discussion

### Statement of principal findings

This systematic review found overall agreement that indicators could play a clear role in motivating physicians to improve the quality of care and showing where changes needed to be made. While it was felt that indicators increased physicians’ accountability, it was clear that they should be used by physicians themselves, rather than by the government or the public, and should not be used punitively. There was concern that an overreliance on indicators might lead to myopic quality improvement at the expense of more holistic care. In order to strengthen the ability of indicators to drive improvements in quality, physicians need to support and participate in the process of indicator development, recording relevant data should be straightforward, indicator feedback needs to be meaningful, and physicians and their teams need to be adequately resourced to act on findings.

While it was recognized that incentives might accelerate quality improvement, there was also the risk of unintended consequences and ‘gaming’. Key attributes of effective indicators were a focus on the most important areas for quality improvement, consistency with good medical care, measurement of aspects of care that were within the control of physicians and reliability. While there was support for the use of patient-reported outcome measures alongside clinical indicators, there was a potential disconnect between the supposed subjectivity of these measures and the desire for indicators to be ‘accurate’ or objective.

### Strengths and limitations

This thematic synthesis of data identified from a systematic review of the literature was focused on physicians’ views regarding the utility of clinical indicators in practice. This is important to understand given the increasing use of clinical indicators and expectations that physicians will use and act on clinical indicator data. As we did not have access to the raw data from primary studies, our findings represent a synthesis of selected data included in the primary studies as well as the authors’ interpretations of that data. The literature search and coding were performed by one reviewer, which may have resulted in bias in the selection of articles. Lastly, texts in languages other than English were excluded.

There were also several limitations in the literature included in this systematic review. As noted, most (9/14) articles did not discuss ethical issues associated with their research with many failing to report ethics approval of the data collection. Generalizability of the results to all physicians is difficult to ascertain because most participants were primary care physicians. Generalizability of the results may also depend on when the data were obtained (given that perspectives are likely to change over time) and the specific health systems examined in each study. Unfortunately, it was not feasible to disaggregate themes according to study context due to the limited number of included studies.

### Interpretation within the context of the wider literature

While our literature search did not return results for physicians’ perspectives on the best tools for appraising the quality of clinical indicators, Jones *et al*. [8] have previously developed the quality improvement critical appraisal tool (QICA), to provide an explicit basis for clinical indicator selection. The findings of our review are consistent with key aspects of the QICA tool, including the need for indicators to measure the most important aspects of medical care and to be evidence-based, acceptable, concordant with other measures of the issue, reliable and to consider the potential for unintended effects, such as bias as well as the resource implications of measurement itself [12]. There were several technical characteristics listed in the QICA tool that were not explored in our systematic review, including the need for a well-defined target population, exclusions and measurement systems, need for indicators to reflect differing cultural values, the power and precision of an indicator to detect clinically important changes beyond random variation, and potential ethical issues involved with data gathering and reporting of results [12].

The final part of the QICA tool addresses the practical implications of indicator implementation in both data collection and data analysis [12]. There was significant overlap here between the characteristics included in the tool and those that physicians thought were important in the systematic review. Similar findings included the importance of limiting extra work in collecting data for clinical indicators, ensuring that technology is sufficient, ensuring that indicator feedback is actionable and that the results are understandable by physicians so they can be used to improve the quality of care.

### Implications for policy, practice, and research

This review found that indicators can play an important motivating role for physicians to improve the quality of care and show where changes need to be made. For indicators to be effective, physicians should be involved in indicator development, recording relevant data should be straightforward, indicator feedback must be meaningful to physicians, and clinical teams need to be adequately resourced to act on findings. Effective indicators need to focus on the most important areas for quality improvement, be consistent with good medical care, and measure aspects of care within the control of physicians. Studies cautioned against using indicators primarily as punitive measures, and there were concerns that an overreliance on indicators could lead to a narrowed perspective on quality of care.

In this systematic review, we found that physicians believe that they should participate in the development of indicators and control the use of those indicators. However, it is worth noting that there are other legitimate groups and stakeholders that also have an interest in the development and use of indicators. Physicians form one professional group among a broader range of multi-disciplinary health providers, as well as patients themselves, whose perspectives need to be engaged in indicator development. It has also been argued that a key impediment faced by collaborative healthcare teams working towards quality improvement is the ‘structured embeddedness of medical dominance’ [13]. Balancing the perspectives of multiple professional groups as well as patients while avoiding the tendency for physicians to disengage from the process entirely is one of the challenges for the use of clinical indicators for driving quality improvements in policy as well as practice, and would be a valuable area for future research.

## Conclusions

This review identified facilitators and barriers to meaningfully engaging physicians in developing and using clinical indicators to improve the quality of healthcare. Such information will help maximize the extent to which the potential of ‘big data’ in revolutionizing clinical engagement with quality improvement activites is able to be realized.

## Supplementary Material

mzae082_Supp

## Data Availability

No new data were generated or analysed in support of this research.
