# Supplementary material for: Physicians’ perspectives on clinical indicators: systematic review and thematic synthesis
Source: Int J Qual Health Care. 2024 Aug 10;36(3):mzae082. doi: 10.1093/intqhc/mzae082 (PMC11369353; doi:10.1093/intqhc/mzae082)
Supplement: mzae082_Supp [file mzae082_supp.zip › suppl_data/Clinical indicators systematic review_Supplementary data_9 Apr 2024.docx]

## **Supplementary material**

**Appendix 1** Search terms by database

| **Medline** | | | |
| --- | --- | --- | --- |
| Search terms |  |  |  |
|  | AND | AND | AND |
| Term from research questions | *Physician* | *Perspective* | *Clinical indicators* |
| Subject headings | Physician | Attitude | Quality Indicators, Health Care |
|  |  | Perception |  |
|  |  |  |  |
| Text words | Physician* | Attitude* | Quality indicator* |
|  | Doctor* | Perception* | Clinical indicator* |
|  | Clinician* | Perspective* |  |
|  |  | View* |  |
|  |  |  |  |
| Subject headings | Attitude of health personnel | |  |
|  |  | |  |
| Text words | Attitude* of health personnel | |  |
|  | Health personnel attitude* | |  |
| *Truncation symbol used to search databases with variation in word endings  Subject headings not exploded | | | |

| **Embase** | | | |
| --- | --- | --- | --- |
|  | AND | AND | AND |
| Terms from research questions | *Physician* | *Perspective* | *Clinical indicators* |
| Subject headings | Physician | Attitude | Clinical Indicator |
|  | Clinician | Perception |  |
|  |  |  |  |
| Text words | Physician* | Attitude* | Clinical indicator* |
|  | Doctor* | Perception* | Quality indicator* |
|  | Clinician* | Perspective* |  |
|  |  | View* |  |
|  |  |  |  |
| Subject headings | Health personnel attitude | |  |
|  | Physician attitude | |  |
|  |  | |  |
| Text words | Health personnel attitude* | |  |
|  | Attitude* of health personnel | |  |
| *Truncation symbol used to search databases with variation in word endings  Subject headings not exploded | | | |

| **Scopus** |  |  |  |
| --- | --- | --- | --- |
|  | AND | AND | AND |
| Terms from research questions | *Physician* | *Perspective* | *Clinical indicators* |
| Text words | Physician* | Attitude* | “Clinical indicator*” |
|  | Doctor* | Perception* | “Quality indicator*” |
|  | Clinician* | Perspective* |  |
|  |  | View* |  |
|  |  |  |  |
| Text words | “Health personnel attitude*” | |  |
|  | “Attitude* of health personnel” | |  |
| *Truncation symbol used to search databases with variation in word endings  Subject headings not exploded | | | |

| **Cochrane Library** | | | |
| --- | --- | --- | --- |
|  | AND | AND | AND |
| Terms from research questions | *Physician* | *Perspective* | *Clinical indicators* |
| Subject headings | Physicians | Attitude | “Quality indicators, Health care” |
|  |  | Perception |  |
|  |  |  |  |
| Text words | Physician* | Attitude* | “Quality indicator*” |
|  | Doctor* | Perception* | “Clinical indicator*” |
|  | Clinician* | Perspective* |  |
|  |  | View* |  |
|  |  |  |  |
| Subject headings | Attitude of health personnel | |  |
|  |  | |  |
| Text words | “Attitude* of health personnel” | |  |
|  | “Health personnel attitude*” | |  |
| *Truncation symbol used to search databases with variation in word endings  Subject headings not exploded | | | |

| **CINAHL** | | | |
| --- | --- | --- | --- |
|  | AND | AND | AND |
| Terms from research questions | *Physician* | *Perspective* | *Clinical indicators* |
| Subject headings | Physicians | Attitude | Clinical indicators |
|  |  | Perception |  |
|  |  |  |  |
| Text words | Physician* | Attitude* | “Quality indicator*” |
|  | Doctor* | Perception* | “Clinical indicator*” |
|  | Clinician* | Perspective* |  |
|  |  | View* |  |
|  |  |  |  |
| Subject headings | Attitude of health personnel | |  |
|  | Physician attitudes | |  |
|  |  | |  |
| Text words | “Attitude* of health personnel” | |  |
|  | “Health personnel attitude*” | |  |
| *Truncation symbol used to search databases with variation in word endings  Subject headings not exploded | | | |

| **PsycInfo** | | | |
| --- | --- | --- | --- |
|  | AND | AND | AND |
| Terms from research questions | *Physician* | *Perspective* | *Clinical indicators* |
| Subject headings | Physicians | Attitudes |  |
|  | Clinicians | Perception |  |
|  |  |  |  |
| Text words | Physician* | Attitude* | Quality indicator* |
|  | Doctor* | Perception* | Clinical indicator* |
|  | Clinician* | Perspective* |  |
|  |  | View* |  |
|  |  |  |  |
| Subject headings | Health personnel attitudes | |  |
|  |  | |  |
| Text words | Attitude* of health personnel | |  |
|  | Health personnel attitude* | |  |
| *Truncation symbol used to search databases with variation in word endings  Subject headings not exploded | | | |

| **Web of Science Core Collection** | | | |
| --- | --- | --- | --- |
|  | AND | AND | AND |
| Terms from research questions | *Physician* | *Perspective* | *Clinical indicators* |
| Text words | Physician* | Attitude* | “Quality indicator*” |
|  | Doctor* | Perception* | “Clinical indicator*” |
|  | Clinician* | Perspective* |  |
|  |  | View* |  |
|  |  |  |  |
| Text words | “Attitude* of health personnel” | |  |
|  | “Health personnel attitude*” | |  |
| *Truncation symbol used to search databases with variation in word endings  Subject headings not exploded | | | |

| **Appendix 2** Summary of quality assessment of included studies | | | | | | | | | | |
| --- | --- | --- | --- | --- | --- | --- | --- | --- | --- | --- |
| Study (year) | Clear aims | Methodology appropriate | Research design appropriate | Recruitment strategy appropriate | Data collection appropriate | Relationship adequately considered | Ethical issues considered | Data analysis rigorous | Clear findings | Research valuable |
| Ahmed et al. (2019) | Yes | Yes | Yes | Yes | Yes | Unclear | Yes | Yes | Yes | Yes |
| Benn et al. (2015) | Yes | Yes | Yes | Yes | Yes | Yes | Unclear | Yes | Yes | Yes |
| Breidenbach et al. (2021) | Yes | Yes | Yes | Yes | Yes | Unclear | Yes | Yes | Yes | Yes |
| D’Lima et al. (2017) | Yes | Yes | Yes | Yes | Yes | No | Yes | Yes | Yes | Yes |
| Exworthy et al. (2003) | Yes | Yes | Yes | Yes | Yes | No | Unclear | Yes | Yes | Yes |
| Gagliardi et al. (2008) | No | Yes | Yes | Yes | Yes | No | Unclear | Yes | Yes | Yes |
| Gill et al. (2012) | Yes | Yes | Yes | Yes | Yes | Yes | Yes | Yes | Yes | Yes |
| Gray et al. (2018) | Yes | Yes | Yes | Unclear | Yes | No | Unclear | Yes | Yes | Yes |
| Hicks et al. (2021) | No | Yes | Unclear | Unclear | Yes | Unclear | Unclear | Unclear | Yes | Yes |
| Litvin et al. (2015) | Yes | Yes | Yes | Yes | Yes | No | Unclear | Yes | Yes | Yes |
| Maxwell et al. (2002) | Yes | Yes | Yes | Unclear | Yes | Yes | Unclear | Unclear | Yes | Yes |
| Rasooly et al. (2022) | Yes | Yes | Unclear | Yes | Yes | Unclear | Unclear | Yes | Yes | Yes |
| Van den Heuvel et al. (2010) | Yes | Yes | Yes | Yes | Yes | No | No | Yes | Yes | Yes |
| Wilkinson et al. (2000) | Yes | Yes | Yes | Yes | Yes | No | Unclear | Yes | Yes | Yes |
